# Single Agent and Synergistic Activity of Maritoclax with ABT-263 in Nasopharyngeal Carcinoma (NPC) Cell Lines

**DOI:** 10.21315/tlsr2020.31.3.1

**Published:** 2020-10-15

**Authors:** Benedict Lian Shi Xiang, Lo Kwok-Wai, Alan Khoo Soo-Beng, Nethia Mohana-Kumaran

**Affiliations:** 1 School of Biological Sciences, Universiti Sains Malaysia, 11800 USM Pulau Pinang, Malaysia; 2 Department of Anatomical and Cellular Pathology and State Key Laboratory in Oncology in South China, The Chinese University of Hong Kong, Central Ave, Hong Kong; 3 Molecular Pathology Unit, Cancer Research Centre, Institute for Medical Research, Jalan Pahang, 50588 Kuala Lumpur, Malaysia

**Keywords:** Nasopharyngeal Carcinoma, 3D NPC Spheroids, Maritoclax, ABT-263, BH3 Mimetics, Kanser Nasofarinks, Sferoid 3D Kanser Nasofarinks, Maritoclax, ABT-263, BH3 Mimetik

## Abstract

The BCL-2 anti-apoptotic proteins are over-expressed in many cancers and hence are attractive therapeutic targets. In this study, we tested the sensitivity of two Nasopharyngeal Carcinoma (NPC) cell lines HK1 and C666-1 to Maritoclax, which is reported to repress anti-apoptotic protein MCL-1 and BH3 mimetic ABT-263, which selectively inhibits anti-apoptotic proteins BCL-2, BCL-XL and BCL-w. We investigated the sensitisation of the NPC cell lines to these drugs using the SYBR Green I assay and 3D NPC spheroids. We report that Maritoclax repressed anti-apoptotic proteins MCL-1, BCL-2, and BCL-XL in a dose- and time-dependent manner and displayed a single agent activity in inhibiting cell proliferation of the NPC cell lines. Moreover, combination of Maritoclax and ABT-263 exhibited synergistic antiproliferative effect in the HK1 cells. Similar results were obtained in the 3D spheroids generated from the HK1 cells. More notably, 3D HK1 spheroids either treated with single agent Maritoclax or combination with ABT-263, over 10 days, did not develop resistance to the treatment rapidly. Collectively, the findings illustrate that Maritoclax as a single agent or combination with BH3 mimetics could be potentially useful as treatment strategies for the management of NPC.

## Abstract (ms)

Protein anti-apoptotik BCL-2 diekspres pada tahap yang tinggi dalam kebanyakan kanser dan merupakan sasaran terapeutik kanser yang menarik. Dalam kajian ini, sensitiviti dua titisan sel kanser nasofarinks diuji, iaitu HK1 dan C666-1 kepada Maritoclax yang dilaporkan menurunkan tahap protein anti-apoptotik MCL-1 dan BH3 mimetik ABT-263 yang merencat protein anti-apoptotik BCL-2, BCL-XL dan BCL-w. Sensitiviti sel kanser nasofarinks kepada dadah ini disiasat menggunakan ujian SYBR Green I dan sferoid 3D kanser nasofarink. Di sini kami melaporkan bahawa Maritoclax mengurangkan tahap MCL-1, BCL-2 dan BCL-XL mengikut konsentrasi *drug* dan tempoh rawatan dan Maritoclax memaparkan aktiviti agen tunggal dalam menghalang percambahan sel kanser nasofarinks. Selain itu, gabungan Maritoclax dan ABT-263 menghalang percambahan sel kanser nasofarinks HK1 secara sinergistik. Keputusan yang sama diperolehi dengan sferoid 3D sel HK1. Lebih penting, sferoid 3D sel HK1, sama ada dirawat dengan agen tunggal Maritoclax atau gabungan dengan ABT-263 selama 10 hari, tidak menunjukkan resistan terhadap rawatan dengan cepat. Secara kolektif, kajian ini menunjukan bahawa Maritoclax sebagai ejen tunggal atau gabungan dengan BH3 mimetik boleh menjadi strategi rawatan yang berpotensi untuk NPC.

**Highlights**

- Maritoclax inhibited cell proliferation of NPC cell lines HK1 and C666-1 as a single agent in a dose-dependent manner in 2-dimensional and 3-dimensional cell culture models.
- Besides repressing MCL-1 in a dose- and time-dependent manner, Maritoclax repressed BCL-XL modestly in a dose- and time-dependent manner and transiently repressed BCL-2 in C666-1 cells in a dose-dependent manner but exhibited a strong time-effect on the level of BCL-2.
- We demonstrated with appropriate drug concentrations, Maritoclax, could be used as a sensitiser to ABT-263. More notably, the combination did not result rapid development of resistance in 3D NPC spheroids.

## INTRODUCTION

Malaysia has one of the highest national incidences of Nasopharyngeal Carcinoma (NCP) in Southeast Asia (Bray *et al*. 2018). Treating patients with metastatic NPC is often a challenge as patients develop resistance to systemic anti-cancer therapies such as chemotherapy and retreating local recurrence with radiotherapy have many limitations. Thus, novel or improved treatment strategies are urgently needed to curb this disease.

The BCL-2 family proteins are divided into pro- and anti-apoptotic proteins. They are critical regulators of the intrinsic apoptosis pathway (Montero & Letai 2018). The anti-apoptotic BCL-2 proteins are overexpressed in many cancers and thus have become attractive therapeutic targets especially with the development of BH3-mimetics such as ABT-263 which selectively targets these proteins. There are number of studies which have reported on the expressions of the anti-apoptotic proteins in NPC tissues. BCL-2 expression was detected in 80% NPC tissues and 71% adjacent dysplastic lesions compared to normal nasopharynx epithelia, using the immunohistochemistry (IHC) technique (Sheu *et al*. 1997). Employing the same technique, similar results were obtained in other studies (Chen *et al*. 2010; Fan *et al*. 2006; Yu *et al*. 2003). NPC tissues positive for BCL-2 expression were highly correlated with neck lymph nodes metastasis (Chen *et al*. 2010) and a worse disease-free 5-year survival (Chen *et al*. 2008). Collectively, these studies suggest that the BCL-2 anti-apoptotic proteins are relevant targets for NPC treatment.

ABT-263 a small molecule drug which exhibits similar bin binds with high affinity to anti-apoptotic proteins BCL-2 and BCL-XL and with a lower affinity to BCL-w (Leverson *et al*. 2015; Tse *et al*. 2008). The drug has exhibited single agent activity against hematological malignancies and small cell lung cancer (SCLC). However, most solid tumours are resistant to treatment of ABT-263. It has become evident that sensitivity to ABT-263 is determined by anti-apoptotic protein MCL-1. Thus, inhibition of MCL-1 or induction of NOXA (antagonist of MCL-1) have shown to sensitise solid cancer cells to ABT-263 (Airiau *et al*. 2016; Jane *et al*. 2016; Lucas *et al*. 2012; Nakajima *et al*. 2016). Maritoclax a natural compound identified from a species of marine-derived streptomycetes (Hughes *et al*. 2008; 2009) was reported to antagonise MCL-1 and target the protein for proteasome-mediated degradation (Doi *et al*. 2012). Moreover, Maritoclax was reported to sensitise a number of cancer cell lines to ABT-737 (first generation BH3 mimetic which inhibits BCL-2, BCL-XL and BCL-w) by down-regulating MCL-1 (Doi *et al*. 2012). In the present study, we investigated the sensitivity of two NPC cell lines HK1 and C666-1 to single agent treatment of Maritoclax and ABT-263 alone and in combination using 2-dimensional (2D) and 3-dimensional (3D) cell culture models.

## MATERIALS AND METHODS

### Cells and Cell Culture

The human NPC cell lines HK1 and C666-1 were grown and authenticated using the AmpFISTR profiling as described in published literature (Daker *et al*. 2012). Experiments were performed within 2–3 cell passages of the foundation stocks.

### Western Blot Analysis

Cells were lysed and analysed on reducing SDS-PAGE as described in published literature (Lucas *et al*. 2012). Membranes were blocked with 5% blotto and probed with antibodies against: MCL-1 (clone 22, BD Pharmingen, USA), BCL-2 (clone 100, BD Pharmingen, USA), BCL-XL (clone 2H12, BD Pharmingen, USA) and α-tubulin (Y1/2, Abcam, UK). Membranes were washed with phosphate-buffered saline with Tween® (PBST) several times and bound antibodies were detected using either goat anti-mouse IgG-HRP (sc-2005, Santa Cruz Biotechnology, USA) or goat anti-rat IgG-HRP (sc-2006, Santa Cruz Biotechnology, USA) and enhanced with Super Signal® West Pico Chemiluminescent (Thermo Scientific, USA).

### SYBR Green I Assay and Synergy Analysis

The SYBR Green I assay was conducted as described in published literature (Allen *et al*. 2002; McGowan *et al*. 2011). The HK1 cells were seeded at a density of 2500 cells/well and the C666-1 cells were seeded at a density of 4000 cells/ well in 96-well plates and left to attach for 6 to 7 hours. Cells were treated to a concentration series [0–32 μM] of single agent Maritoclax (ChemScene, USA) and ABT-263 (Selleckchem, USA) and as combination along the long plate axis for 72 h. Sensitisation to ABT-263 by Maritoclax was assessed by testing a fixed concentration of Maritoclax to increasing concentrations and ABT-263 for 72 h. Cell proliferation was quantified as described in published literature (Lian *et al*. 2018).

### Test of Synergy

Drug synergy analyses were performed using the median effect principal as previously described by Chou and Talalay (Chou 2006; Chou & Talalay 1984). The CalcuSyn 2.11 software (Biosoft, UK) was employed to produce the Fa-Cl isobologram plots and the combination index (CI) values, which is a statistical measure of synergy. CI values < 1 indicate synergy, = 1 indicate an additive effect and > 1 indicate antagonism, CI values nearing 0 indicate strong synergy.

### Generation of 3D Spheroids

Approximately 5000 (2.5 × 10^4^ cells mL^−1^) HK1 cells were seeded in the ultra-low attachment (ULA) 96-well U bottom plate (Corning, USA) and centrifuged at 1200 rpm for 2 min. The plates were incubated in a humidified incubator at 37°C with 5% CO_2_ for three days. Spheroids were embedded in collagen matrix as described (Smalley *et al*. 2006; 2008). Spheroids were treated with ABT-263 and Maritoclax at drug doses in 1 mL of complete medium and were incubated in a humidified incubator at 37°C with 5% CO_2_. Phase contrast snapshots of spheroids were taken every 24 h using the IX71 Olympus inverted fluorescence microscope over 10 days to document spheroid growth and invasion. Spheroids were washed three times in phosphate-buffered saline (PBS) and stained with 4 mmol L^−1^ calcein AM and 2 mmol L^−1^ ethidium homodimer I (Thermo Fisher Scientific, USA) for 1 h at 37°C. Images of spheroids were captured using the IX71 Olympus inverted fluorescence microscope.

## RESULTS

### Maritoclax Suppresses BCL-2 Anti-Apoptotic Proteins in a Dose- and Time-Dependent Manner

**Figure 1:**
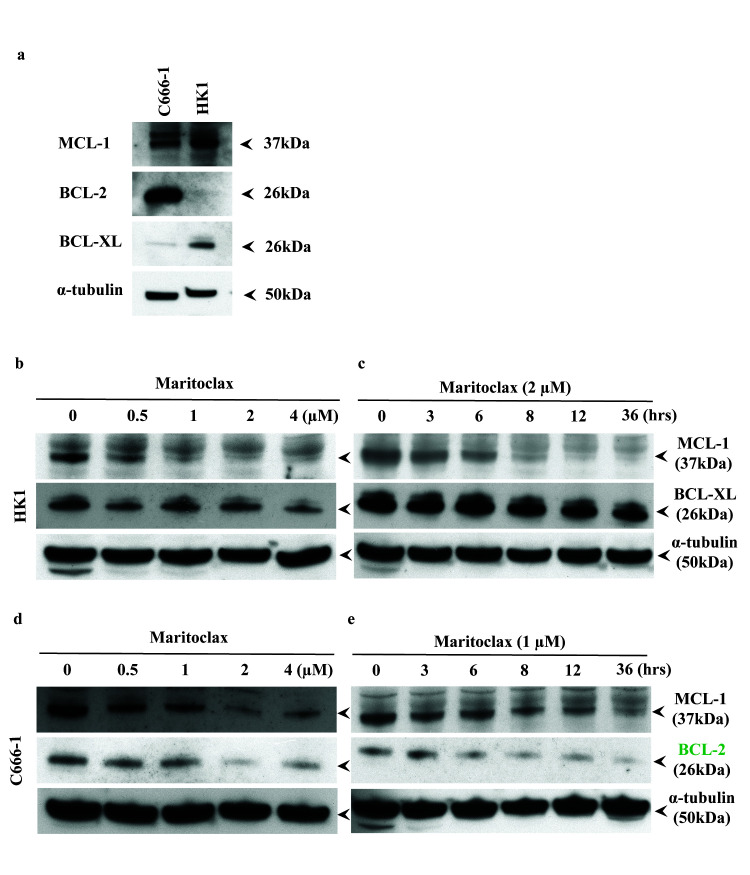
Maritoclax repressed the anti-apoptotic proteins in a dose- and time-dependent manner. (a) Basal expression levels of the anti-apoptotic proteins in the NPC cell lines. The basal expression levels of MCL-1, BCL-2 and BCL-XL in the C666-1 and the HK1 cells were determined by SDS-PAGE gel electrophoresis. NPC cell lines HK1 and C666-1 cells were treated with (b, d) Escalating doses of Maritoclax for 24 h to study the dose response or (c, e) with 2 μM or 1 μM of Maritoclax in the HK1 and C666-1 cells, respectively, at the indicated time points to study the time response. Immunoblot analyses illustrated that repression of MCL-1 by Maritoclax in both cell lines were dose- and time-dependent. (b–c) Maritoclax demonstrated a modest dose and time effect on the expression level of BCL-XL in the HK1 cells. (d–e) Maritoclax had a transient dose effect but a strong time effect on the level of BCL-2 in the C666-1 cells. Alpha-tubulin was used as the loading control. Arrows indicate the protein bands probed on the x-ray films.

The basal expression levels of the anti-apoptotic proteins MCL-1, BCL-XL and BCL-2 in the NPC cells were first investigated. MCL-1 was expressed in both HK1 and C666-1 cells (Fig. 1a). High expression of BCL-2 was found in the C666-1 cells, whereas relatively high expression of BCL-XL was found in the HK1 cells (Fig. 1a).

Next, we determined the effect of Maritoclax on MCL-1 protein level. The HK1 and C666-1 cells were treated with Maritoclax at different doses and time points. Maritoclax reduced MCL-1 protein level in both cell lines at concentration as low as 0.5 μM. The expression level of MCL-1 continued to reduce as the drug concentration increased (Figs. 1b and d). Maritoclax also resulted in a time-dependent, reduction of MCL-1. In both cell lines reduction of MCL-1 was obvious at 8 h and continued to reduce with time (Figs. 1c and 1e). Hence, the net effect of Maritoclax on MCL-1 protein level was both time- and dose-dependent.

The effect of Maritoclax on the expression levels of BCL-XL and BCL-2 were also investigated. In the HK1 cells, the level of BCL-XL and not BCL-2 was investigated, as the basal level of BCL-2 in this cell line was too low (Fig. 1a) and *vice versa* in the C666-1 cells. Treatment of HK1 cells with Maritoclax repressed BCL-XL modestly in a dose- and time-dependent manner (Figs. 1b and 1c). Interestingly, in the C666-1 cells, increasing concentrations of Maritoclax exhibited a transient dose effect on the level of BCL-2 (Fig. 1d). The reduction of BCL-2 was near complete at 2 μM of Maritoclax but at 4 μM of Maritoclax, the protein level was restored (Fig. 1d). However, Maritoclax exhibited a strong time-effect on the expression level of BCL-2 in the C666-1 cells (Fig. 1e). The ability of Maritoclax to repress the other anti-apoptotic proteins indicates that it is not a selective MCL-1 inhibitor.

### NPC Cells Sensitive to Single Agent Treatment of Maritoclax in a Dose-Dependant Manner

**Figure 2:**
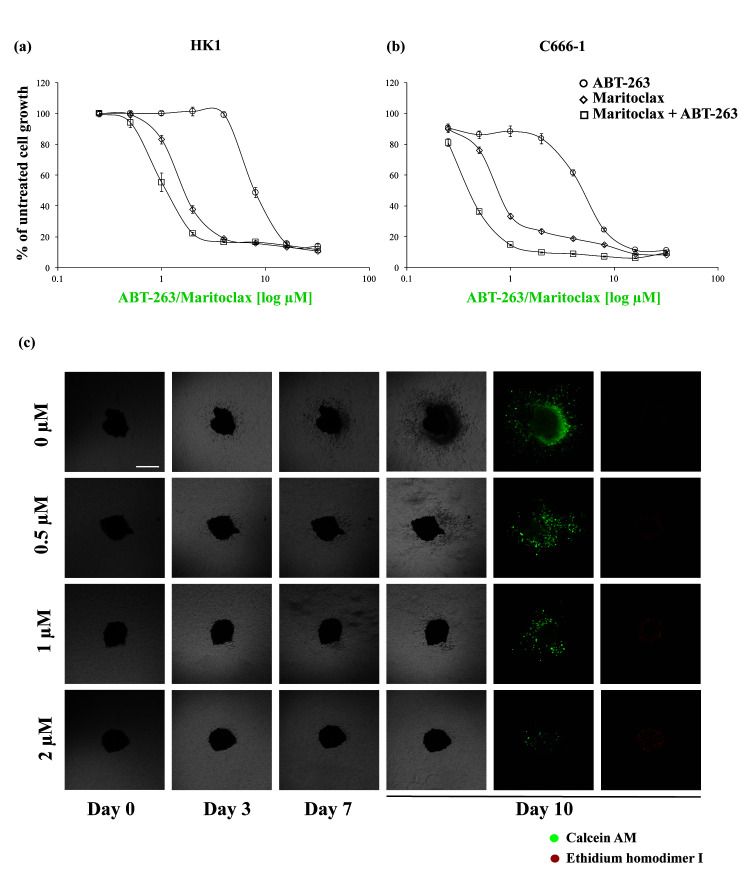
Maritoclax exhibited single agent activity in inhibiting cell proliferation of the NPC cells. (a) HK1 cells and (b) C666-1 cells were treated with increasing concentrations of ABT-263 (0–32 μM) (open circle) or Maritoclax (0–32 μM) (open diamond) or combination of ABT-263 and Maritoclax (open square) at 1:1 drug concentration ratio for 72 h. Cell proliferation was assessed using the SyBr Green I assay. Points represent mean ± SEM of four experiments. (c) One representative experiment showing HK1 spheroids demonstrating decrease viable cells (calcein-AM) and increase dead cells (Ethidium homodimer I) after treatment with single agent Maritoclax at increasing concentrations over 10 days. Medium and drug were replenished every 72 h. Size bar: 500 μM.

The sensitivity of the HK1 and C666-1 cells were first tested to single agent Maritoclax and ABT-263 at increasing concentrations. Unexpectedly treatment with Maritoclax resulted in a profound shift of the dose-response curve to the left indicating a single agent activity of Maritoclax in inhibiting cell proliferation of both cell lines (Figs. 2a and 2b – open diamond). Combination of Maritoclax with ABT-263 at 1:1 drug concentration only resulted in a minimal shift of the dose-response curves in both cell lines (Figs. 2a and 2b – open square).

Similar to the 2D findings, the spheroids established from the HK1 cells were sensitive to single-agent Maritoclax, reflected in dose-dependent inhibition of invasion as well as decreased viable cell staining (Fig. 2c – calcein-AM staining) and increased dead cell staining (Fig. 2c – ethidium homodimer I). More notably there were no resistance cells invading the matrix over 10 days.

### Maritoclax and ABT-263 Synergistically Inhibit Cell Proliferation of the HK1 Cells in 2D and 3D Cell Culture Models

**Figure 3:**
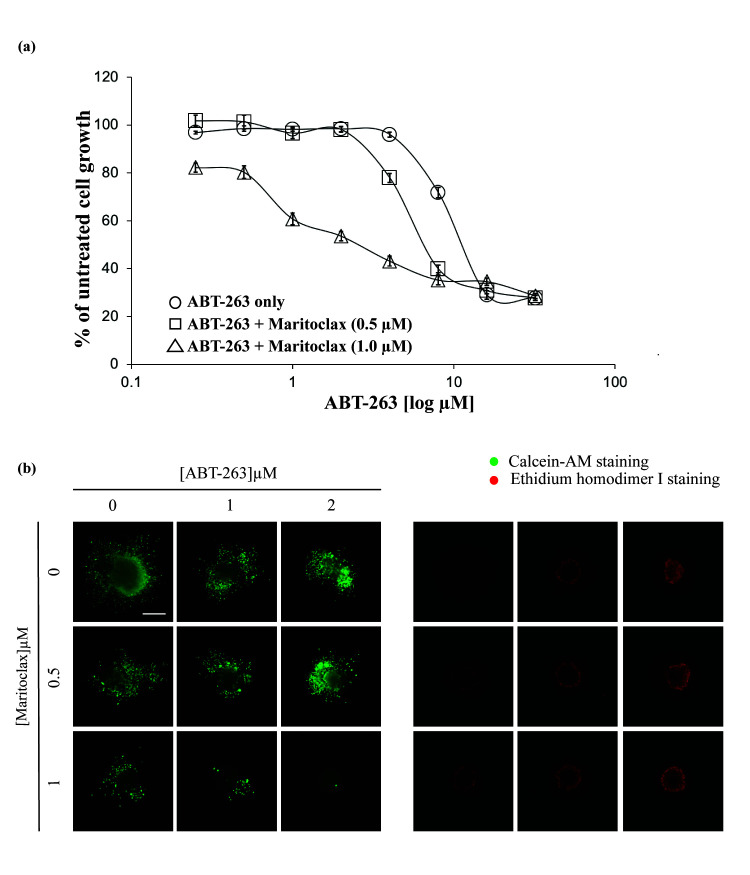
Synergistic anti-proliferative effects of ABT-263 and Maritoclax in the HK1 cells. (a) HK1 cells were treated with increasing concentrations of ABT-263 (0–32 μM) in the presence or absence of either 0.5 or 1 μM of Maritoclax for 72 h. Cell proliferation was assessed using the SyBr Green I assay. Points represent mean ± SEM of four experiments. (b) One representative experiment showing HK1 spheroids demonstrating decrease viable cells (calcein-AM) and increase dead cells (Ethidium homodimer I) after treatment with single agent Maritoclax and ABT-263 and combination of both drugs at increasing concentrations over 10 days. Size bar: 500 μM.

Next, we tested the sensitivity of the HK1 cells to combination of ABT-263 and Maritoclax. HK1 cells were treated with either fixed dose of 0.5 μM or 1 μM of Maritoclax and increasing concentrations of ABT-263 (0–32 μM) (Fig. 3a). The fixed doses of Maritoclax used, were below the single agent IC_50_ value of Maritoclax obtained for HK1 cells (Fig. 2a – open diamond). Concentration of Maritoclax of 0.5 μM exhibited only a 1.7-fold sensitisation of the HK1 cells to ABT-263. Sensitivity of the cells increased to 4-fold when concentration of Maritoclax was increased to 1 μM (Table 1). The mode of interactions of the drug combinations were analysed using the CompuSyn 1.0 software (ComboSyn Inc. NJ, USA). The CI values obtained for combination of Maritoclax and ABT-263 exhibited synergism at several concentrations in the HK1 cell line (Table 2).

The spheroids generated from the HK1 cells were also sensitised to ABT-263 by Maritoclax. The spheroids were treated with ABT-263 and Maritoclax, either alone or in combination over 10 days with medium and drugs replenishment every 72 h. In the presence of 1 μM Maritoclax, there was obvious sensitisation to ABT-263 which reflected in dose-dependent inhibition of spheroid invasion. This also manifested as reduced cell viability (decreased Calcein AM staining and increased Ethidium homodimer I staining) (Fig. 3b).

## DISCUSSION

Given that Maritoclax promote proteasomal degradation of MCL-1 (Doi *et al*. 2012; 2014), there was a reason to believe that combination with ABT-263 may overcome resistance and sensitise the NPC cell lines to ABT-263. Interestingly, a number of studies reported that Maritoclax did not display a stringent selective cytotoxicity on MCL-1 dependent cell lines (Eichhorn *et al*. 2013). Human leukemia cell line RS4;11 which is dependent on BCL-2 for survival was more sensitive to Maritoclax treatment compared to HeLa cells which are dependent on MCL-1 for survival. Furthermore Maritoclax treatment did not alter the expression of MCL-1 in both these lines (Eichhorn *et al*. 2013). In another study, Maritoclax was shown to repress MCL-1 but this effect was not long lasting as the expression level of MCL-1 was restored at later time points in the MCL-1 dependent H460 cells. However, the drug did not repress the levels of anti-apoptotic proteins BCL-2, BCL-XL and BCL-w in the H460 cells (Varadarajan *et al*. 2015). In contrast, Dinaciclib a broad spectrum cyclin-dependent kinase (CDK) inhibitor which was reported to down-regulate MCL-1 exhibited a lasting time effect on the level of MCL-1 in the H460 cells (Varadarajan *et al*. 2015). The same study reported that the treatment with Maritoclax led to more death of the MCL-1 deficient mouse embryonic fibroblasts (MEFs) compared to wild-type MEFs indicating that apoptosis can be triggered via other mechanisms (Varadarajan *et al*. 2015). In our hands, besides repressing MCL-1, Maritoclax exhibited a modest/transient dose effect on the expression levels of BCL-XL and BCL-2 in the NPC cell lines. Moreover, Maritoclax displayed a modest time effect on the expression level of BCL-XL in the HK1 cells and a strong time-effect on the expression level of BCL-2 in the C666-1 cell line indicating that the effect of Maritoclax on the expression levels of other anti-apoptotic proteins could be cell-type specific. The ability of Maritoclax to repress more than one anti-apoptotic protein may explain the single agent activity of Maritoclax in both the NPC cell lines as repression of MCL-1 and BCL-2/BCL-XL may be sufficient to reduce the threshold for apoptosis activation. Similar single agent activity of Maritoclax was reported in melanoma cells (Pandey *et al*. 2013).

There were a number of studies, which reported synergy between Maritoclax and ABT-263 in leukemia and melanoma cells *in vitro* (Doi *et al*. 2012; 2014; Pandey *et al*. 2013). Treatment with suboptimal concentration of Maritoclax (2 μM) repressed MCL-1 and markedly sensitised melanoma cell line UACC903 to ABT-263 by ~23-fold. Sensitisation of the melanoma cells was also evident in the 3D melanoma spheroids (Pandey *et al*. 2013). Combination of ABT-737 with sub-lethal dose of 2 or 2.5 μM Maritoclax in the K562 or Raji cells sensitised the cells to ABT-737 by ~60- and 2000-fold, respectively (Doi *et al*. 2012). Similarly, two acute myeloid cells (AML) namely HL60 and KG1a, which were resistant to ABT-737 due to prolonged culture with the drug, were sensitised to ABT-737 after combination with sub-lethal dose of 2 or 1 μM Maritoclax, respectively (Doi *et al*. 2014). In our study we had to be more cautious with the concentrations of Maritoclax used when combined with ABT-263, as Maritoclax was able to inhibit cell proliferation as single agent at very low concentrations (< 2 μM). Treatment with 1 μM of Maritoclax, sensitised the HK1 cells to ABT-263 by 4-fold. Similar findings were obtained in the 3D spheroids. Given that Maritoclax exhibited single agent activity in both cell lines, it appears that treatment with Maritoclax alone will be sufficient to inhibit cell proliferation of NPC cells. Nevertheless, Maritoclax could be used as a sensitiser to sensitise NPC cells and other cancer cells to BH3 mimetics or other drugs if appropriate drug concentrations are applied.

## CONCLUSION

Taken together, our results show that Maritoclax could be a potential therapy for NPC as a single agent or in combination with ABT-263. However, several limitations of this study should be addressed in future work. Given that Maritoclax inhibits BCL-2/BCL-XL, future work should investigate the mechanism behind the suppression of these two proteins by Maritoclax. The possibilities may be that Maritoclax impairs protein translation or transcription of these proteins. Follow-up studies should also investigate the effect of single agent Maritoclax in preclinical models and testing of Maritoclax as a sensitizer to next generation BH3 mimetics and chemotherapeutic drugs for cancer management.

## Figures and Tables

**Table 1:** Sensitisation of HK1 cells to ABT-263 by Maritoclax.

Maritoclax (μM)	ABT-263 IC_50_ ± SEM (μM)	Fold sensitisation by Maritoclax
0	11.44 ± 0.55	
0.5	6.72 ± 0.25	1.7
1	2.84 ± 0.72	4.0

**Table 2:** The combination index (CI) values and the description of the drug combination interactions for sensitisation of HK1 cells to ABT-263 by Maritoclax.

Maritoclax (μm)	ABT-263 (μM)	Combination Index (CI)	Description
1	0.25	0.73	Moderate synergism
	0.5	0.68	Synergism
	1	0.31	Synergism
	2	0.29	Strong synergism
	4	0.27	Strong synergism
	8	0.32	Synergism
